# Quantification of Ki-67 labeling index in pediatric brain tumor immunohistochemistry images

**DOI:** 10.1093/jnen/nlaf163

**Published:** 2026-03-10

**Authors:** Christoforos Spyretos, Juan Manuel Pardo Ladino, Hakon Andersen Blomstrand, Per Nyman, Oscar Snödahl, Alia Shamikh, Nils Elander, Neda Haj-Hosseini

**Affiliations:** Department of Biomedical Engineering, Linköping University, Linköping, Sweden; Center for Medical Image Science and Visualization, Linköping University, Linköping, Sweden; Department of Biomedical Engineering, Linköping University, Linköping, Sweden; Clinical Department of Clinical Pathology, Region Östergötland, Linköping, Sweden; Department of Biomedical and Clinical Sciences, Linköping University, Linköping, Sweden; Center for Medical Image Science and Visualization, Linköping University, Linköping, Sweden; Crown Princess Victoria Children's Hospital, Region Östergötland, Linköping, Sweden; Department of Health, Medicine and Caring Sciences, Linköping University, Linköping, Sweden; Center for Medical Image Science and Visualization, Linköping University, Linköping, Sweden; Department of Health, Medicine and Caring Sciences, Linköping University, Linköping, Sweden; Clinical Department of Radiology in Linköping, Region Östergötland, Linköping, Sweden; Department of Clinical Pathology and Cancer Diagnostics, Karolinska University Hospital, Solna, Sweden; Department of Oncology-Pathology, Karolinska Institute, Solna, Sweden; Department of Biomedical and Clinical Sciences, Linköping University, Linköping, Sweden; Clinical Department of Oncology in Linköping, Region Östergötland, Linköping, Sweden; Department of Biomedical Engineering, Linköping University, Linköping, Sweden; Center for Medical Image Science and Visualization, Linköping University, Linköping, Sweden

**Keywords:** pediatric brain tumor, histopathology, immunohistochemistry, Ki-67 labeling index, deep learning, QuPath, cell segmentation, cell classification, cell density map

## Abstract

Quantification of the Kiel 67 (Ki-67) labeling index (LI) is critical for assessing proliferation and prognosis in tumors but manual scoring remains a common practice. We present an automated framework for Ki-67 scoring in whole slide images (WSIs) developed for research settings using an Apache Groovy code script for QuPath and complemented by a Python postprocessing script that provides cell density maps and summary tables. Tissue segmentation is performed by pixel classifiers and cell segmentation is conducted using StarDist, a deep learning model, followed by adaptive thresholding to classify Ki-67 positive and negative nuclei. The pipeline was applied to a cohort of 632 pediatric brain tumor cases with 734 Ki-67 WSIs from the Children’s Brain Tumor Network. Medulloblastomas showed the highest Ki-67 LI (median: 19.84), followed by atypical teratoid rhabdoid tumors (median: 19.36), brainstem glioma-diffuse intrinsic pontine gliomas (median: 11.50), high-grade gliomas (grades 3, 4) (median: 9.50), and ependymomas (median: 5.88). Lower indices were found in meningiomas (median: 1.84) and the lowest were seen in low-grade gliomas (grades 1, 2) (median: 0.85), dysembryoplastic neuroepithelial tumors (median: 0.63), and gangliogliomas (median: 0.50). The results demonstrate a significant correlation (*P* < .05) in Ki-67 LI across most of the tumor families/types aligning with neuro-oncology and neuropathology consensus.

## INTRODUCTION

In histopathology, hematoxylin and eosin (H&E) staining is the gold standard for morphological assessment of various tissue types including neoplasms. In addition to H&E, pathologists often use antibody-dependent immunohistochemical (IHC) protocols to identify specific molecular alterations, assess expression levels and distribution of various proteins and determine molecular subgroups of tumors.^1^ One such IHC marker is the antigen Kiel 67 protein (Ki-67), which distinguishes between proliferating and nonproliferating cells. Ki-67 is expressed by nuclear staining and detected in every active phase of the cell cycle, whereas it is absent during the temporary phase. In this regard, the ratio of the number of positive cells to the total number of cells, known as the Ki-67 labeling index (LI), is a key component in assessing the character of diverse tumors.^2^^,^^3^ Ki-67 can be particularly valuable in low-resource settings in which molecular examinations that provide more accurate diagnoses are inaccessible.

Given the extensive usage of Ki-67 in oncological management, it has also been widely studied in adult, pediatric, and adolescent central nervous system (CNS) and brain tumors for providing information on malignant potential and prognosis. An increased Ki-67 LI is associated with higher malignancy in gliomas and is used to differentiate between low-grade and high-grade gliomas. In addition, it is a valuable prognostic factor for estimating tumor progression and survival.^4–6^ Regarding glioblastoma patients, the relationship between Ki-67 LI and overall survival, and its role in distinguishing subtypes remain unclear.^5–7^ Furthermore, molecular subgroups and poorer overall survival of adult medulloblastoma are correlated with Ki-67 LI.^8^ For example, anaplastic medulloblastomas exhibited higher Ki-67 LI, whereas nonanaplastic had lower values.^9^ Regarding meningiomas, an increased Ki-67 LI is associated with higher tumor grade and worse survival.^10^^,^^11^ In the pediatric population, correlations have also been observed between ependymoma grades and Ki-67 LI, and between the prognosis of ependymoma and medulloblastoma with Ki-67 LI.^12^^,^^13^

The percentage of the Ki-67 LI is reported to correlate with the proliferation rate and aggressiveness of neoplasms and consequently the survival prognosis.^2^ Despite a straightforward measure of the proliferation index provided by Ki-67 LI, there is an absence of a standardized method for counting stained nuclei and selecting a representative and adequately sized area (hot spot) in possibly heterogeneously proliferating tumors. Therefore, its clinical utility has been constrained, resulting in poor interobserver reproducibility and high rating variability of the LI.^14–17^ While several studies have demonstrated the benefits of digital image analysis for assessing the Ki-67 LI, there are considerable discrepancies about how to count the cells.^5^^,^^18^ The most reported drawback is the risk of counting nonneoplastic cells, such as lymphocytes, microglia, and macrophages, as well as other brown-pigmented signals, such as hemosiderin and hematoidin. Thus, nonneoplastic cells might contribute to the Ki-67 LI and influence the assessment and interpretation. These errors can be solved by having pathologists manually annotate regions of interest or through IHC double staining with Ki-67 and a tumor cell-specific marker but both approaches are labor-intensive and more costly.

Standardizing the concept of tumor areas with Ki-67-stained nuclei is an essential parameter in calculating the LI, and several noncommercial and research tools have been introduced to mitigate the challenge of identifying tumor and nontumor cells. Traditional image analysis pipelines have been developed for automatic cell segmentation and classification,^19^^,^^20^ while deep learning methods have recently emerged as more powerful alternatives.^21–29^ Many of these traditional and deep learning approaches perform Ki-67 quantification at the patch level or within a restricted region size, rather than across the entire whole slide image (WSI). In addition, integration of these tools into user-friendly interfaces and widely used pathology software is essential, making them readily accessible to researchers and clinicians without requiring advanced computer science knowledge.

This study aimed to develop a research tool for automatic scoring of Ki-67 LI in a commonly used software that can analyze relatively large numbers of images without user intervention and to evaluate its performance on pediatric brain tumors. In this regard, Apache Groovy (Java-based syntax) code script for QuPath,^30^ an open-access software that is routinely used for histology analysis in research settings, was developed and evaluated to automate cell counting across multiple pediatric brain tumor families/types using Ki-67 WSIs.

## METHODS

### Data

In this study, the dataset was obtained from the Children’s Brain Tumor Network (CBTN) in 2023.^31^^,^^32^ The dataset was collected from 32 institutions across the United States, Australia, Switzerland, and Italy, including various stained WSIs with H&E being the most common and Ki-67 being one of the most representative IHC staining protocols. Furthermore, the version of the CBTN dataset obtained is based on pre-2021 WHO guidelines, in which some of the brain tumor classifications are no longer used in clinical practice.^33^ Therefore, in accordance with clinicians, subjects with outdated classifications were excluded and the classifications listed in the 2021 WHO guidelines were used to conduct the experiments. The study analyzed the tumor families of low-grade glioma/astrocytoma (grades 1, 2) (LGG) and high-grade glioma/astrocytoma (grades 3, 4) (HGG), and the tumor types of medulloblastomas (MBs), ependymomas (EPs), gangliogliomas (GGs), meningiomas (MENs), atypical teratoid rhabdoid tumors (ATRTs), dysembryoplastic neuroepithelial tumors (DNETs), and brainstem glioma-diffuse intrinsic pontine glioma (DIPG). The left side of 139 slides containing control tissue was cropped to analyze only the right side. In total, 629 subjects were included in the analysis. However, the diagnosis of 3 subjects changed over time and therefore were treated as independent cases, resulting in 632 cases (male/female: 339/293, mean±standard deviation (m±sd) age in years: 10.43±6.55 years) and 734 Ki-67 WSIs, with some cases having more than 1 slide. The left side of 139 slides containing control tissue on the left side was cropped to analyze only the right side. Additionally, tumor descriptor information for each case was extracted from the corresponding magnetic resonance imaging (MRI) dataset with 413 cases classified as initial CNS tumors, 92 as progressive, 40 as recurrent, and 12 as second malignancy, and 104 tumors did not have any descriptor. Among the cases, 29 had 2 tumor descriptors and 1 case had 3 tumor descriptors. It should be noted that there was no one-to-one correspondence in the rest of the cases between the initial CNS and later tumor descriptors. Table 1 summarizes the final number of subjects and WSIs.

**Table 1.** Number of cases and Ki-67 WSIs for each tumor family/type included in the study.

Tumor family/Type	Ki-67		Ki-67 with tumor descriptor	
	No. of cases (male/female)	No. of WSIs	No. of cases (male/female)	No. of WSIs
Low-grade glioma (grades 1, 2) (LGG)	272 (147/125)	309	244 (131/113)	274
High-grade glioma (grades 3, 4) (HGG)	88 (48/40)	103	76 (43/33)	90
Medulloblastoma (MB)	81 (46/35)	86	75 (42/32)	79
Ependymoma (EP)	71 (45/26)	89	59 (37/22)	75
Ganglioglioma (GG)	51 (28/23)	65	41 (23/18)	54
Meningioma (MEN)	24 (13/11)	34	5 (4/1)	5
Atypical teratoid rhabdoid tumor (ATRT)	20 (13/7)	21	18 (12/6)	19
Dysembryoplastic neuroepithelial tumor (DENT)	14 (11/3)	15	1 (1/—)	1
Brainstem glioma-diffuse intrinsic pontine glioma (DIPG)	11 (7/4)	12	9 (7/2)	10
Total	632 (339/293)	734	528 (276/252)	607

Abbreviations: Ki-67 Kiel 67; WSIs, whole slide images.

### Analysis scripts

**Figure 1.**
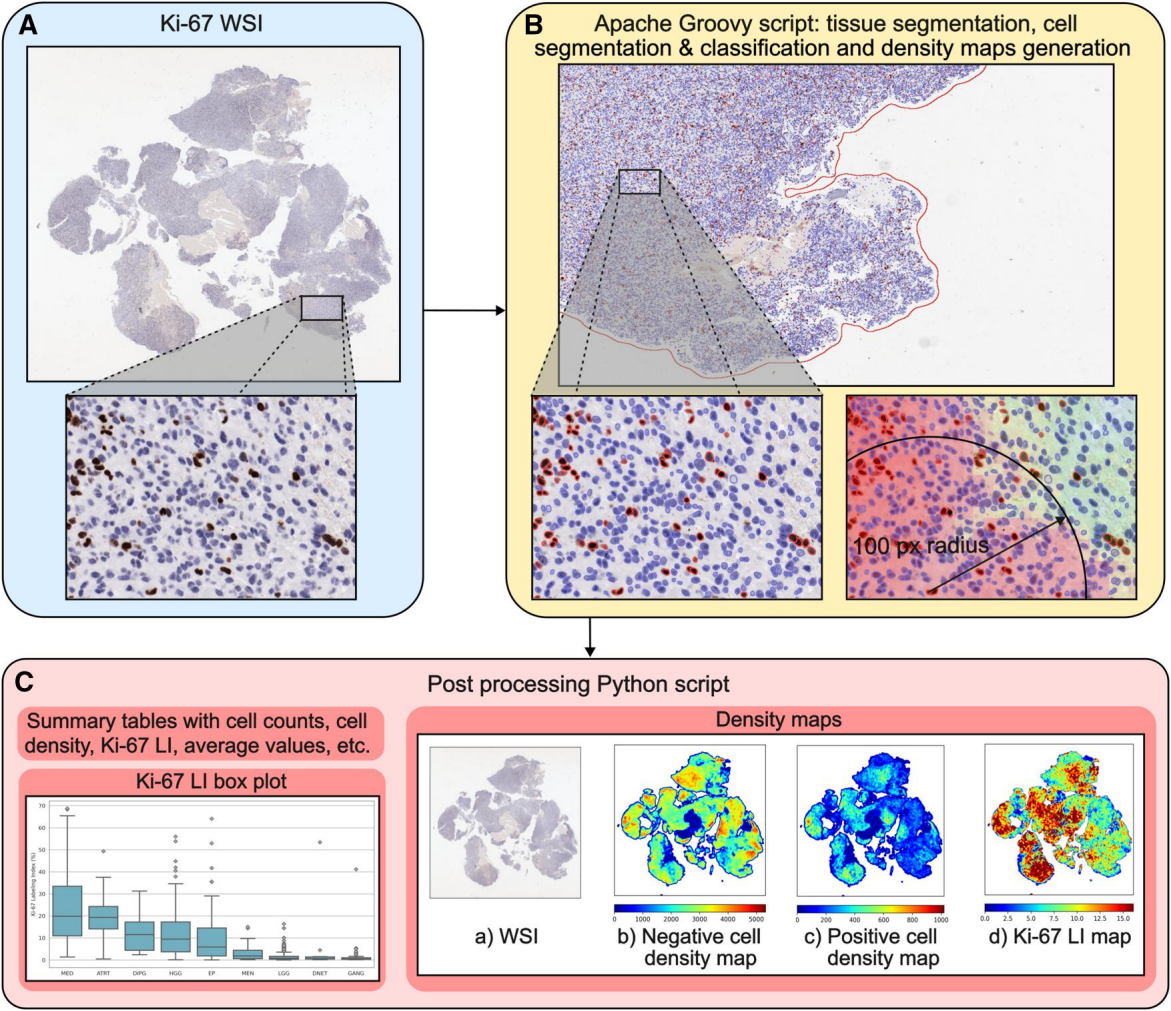
Overview of the analysis workflow. (A) Ki-67 WSIs are imported into QuPath. Representative Ki-67 WSIs with zoomed-in 20 μm region from a subject diagnosed with HGG. Ki-67 negative nuclei are stained blue, and positive nuclei are stained brown. (B) Tissue segmentation, cell segmentation, and classification are performed by the Apache Groovy script. Image metadata and cell density maps are extracted using the classified nuclei with a search radius of 100 pixels (50 μm) and stored. (C) Density maps are processed and summary graphs and tables are produced by the Python script. Abbreviations: HGG, high-grade glioma; Ki-67 Kiel 67; WSIs, whole slide images.

The images’ analysis was performed using an Apache Groovy script in QuPath. A Python script, which is user-friendly and requires minimal coding expertise, was used for postprocessing. After adding all slides at once in QuPath, the Groovy script performed an initial tissue segmentation using a predefined pixel classifier to eliminate artifacts, this was followed by stain vector estimation. In the next step, a threshold-based classifier was applied to achieve refined tissue segmentation based on the updated stain parameters. Subsequently, cell segmentation was performed using the deep learning model StarDist,^24–26^ and cells were classified as positive or negative using an adaptive thresholding method following Lim.^34^ During this process, image metadata was stored and cell density maps are generated. The Python codes produce summary graphs and tables of the Ki-67 LI, which was defined as follows:


$$\mathrm{Ki}-67\;\mathrm{LI}=\frac{\mathrm{Number}\;\mathrm{of}\;\mathrm{positive}\;\mathrm{cells}}{\mathrm{Number}\;\mathrm{of}\;\mathrm{positive}\;\mathrm{cells}+\mathrm{Number}\;\mathrm{of}\;\mathrm{negative}\;\mathrm{cells}}$$

and further process the density maps by normalizing and visualizing them. Users can run the Python script via the terminal by specifying a set of defined arguments. The detailed functions of both scripts are described in the following sections. The analysis workflow is illustrated in Figure 1.

### Apache Groovy script functions

#### Preliminary tissue segmentation

Each WSI was initially set to the Brightfield_H_DAB image type, with defined generic color deconvolution stain vectors. DAB stands for diaminobenzidine, an organic compound used in IHC stains. In addition, a predefined pixel classifier was selected in QuPath, named Base_Classifier, which is applied to eliminate artifacts such as dark areas and pen marks. The values for the minArea and minHoleArea were selected through trial and error to achieve an optimal balance between minimizing noise and capturing tissue regions. The default parameter settings for this preliminary tissue segmentation are outlined in Table 2.

**Table 2.** Default and adapted parameter settings for the pixel classifiers of the preliminary and threshold-based tissue segmentations.

Parameter	Preliminary segmentation	Threshold-based segmentation
Pixel classifier type (default)	OpenCVPixelClassifier	OpenCVPixelClassifier
Input padding	0	0
Input resolution (default)	Pixel width: 4.0016 μm Pixel height: 4.0016 μm Z spacing: 1.0 z-slice	Pixel width: 4.0016 μm Pixel height: 4.0016 μm Z spacing: 1.0 z-slice
Input dimensions (default)	Input width: 512 pixels Input height: 512 pixels Input number of channels: 3 (RGB values)	Input width: 512 pixels Input height: 512 pixels Input number of channels: 3 (RGB values)
Below threshold	—	Tumor (RGB values [200,0,0])
Above threshold	Region (RGB values [0,0,180])	—
Channel (default)	Red	Red
Filter (default)	Gaussian (sigmaX: 8.0, sigmaY: 8.0)	Gaussian (sigmaX: 8.0, sigmaY: 8.0)
Threshold	80 (pixel intensity value>80 are classified as region)	Custom threshold
minArea	20 000 ${\mu m}^{2}$ (minimum area of regions to retain)	10 000 ${\mu m}^{2}$ (minimum area of regions to retain)
minHoleArea	8000 ${\mu m}^{2}$ (the minimum area of connected “hole” regions to retain)	8,000${\;\mu m}^{2}$ (the minimum area of connected “hole” regions to retain)
Options	“DELETE_EXISTING” (delete existing annotations) “INCLUDE_IGNORED” (include ignored objects)	“DELETE_EXISTING” (delete existing annotations)

#### Stain vector estimation

Next, the stain and background vectors were automatically estimated within the segmented region and updated for subsequent analysis. The new stain vectors were calculated using the EstimateStainVectors function from QuPath. First, the image was smoothed to reduce noise and artifacts. Then, the most frequent RGB values were extracted to determine the background color. Finally, the stain vectors were estimated from the smoothed image, computing the new hematoxylin, DAB, and residual stain components.

#### Threshold-based tissue segmentation

In this step, to optimize the preliminary tissue segmentation a custom pixel classifier was used to segment tissue regions by thresholding the red channel of the slide based on the new estimated stain vector values. The threshold value was automatically adapted based on the characteristics of the hematoxylin stain vector for each slide. Specifically, if the hematoxylin vector was greater than or equal to 0.7, indicating a weak stain that complicates tissue foreground segmentation, a higher threshold was automatically applied. In contrast, a lower threshold was used for slides with stronger hematoxylin staining. The adaptive thresholding enabled the classifier to more effectively distinguish foreground tissue from background across slides with varying stain intensities.

Subsequently, the slides with weak hematoxylin staining were automatically flagged, and image-level normalization was applied instead of tile-level normalization during cell segmentation. This adjustment addressed background segmentation issues that could have caused local normalization errors. A new pixel classifier, using the updated custom threshold, was then applied to segment the tissue in the slide, computing the final tissue segmentation. Similarly to the preliminary tissue segmentation, the minArea and minHoleArea values were determined through trial and error to obtain a balance between reducing noise and preserving tissue regions. The parameters of the threshold-based tissue segmentation are summarized in Table 2.

#### Cell segmentation and classification

The cell segmentation was performed using StarDist, a publicly available pretrained deep learning model.^24–26^ More specifically, pixel intensity normalization was performed between the 1st and 99th percentiles of each tile, allowing the model to adapt to the characteristics of each tile. The probability threshold for cell detection was set to 0.25 and the pixel size was specified to 0.5 μm. Lower values did not improve the results, increasing computational demand, and higher values led to less precise segmentation. StarDist is designed to resolve overlapping nuclei by default based on the star-convex polygon nucleus representation combined with the nonmaximum suppression approach.

The cell classification between positive and negative cells was performed by defining an adaptive threshold based on the triangle thresholding method, which considered the color variations in each image. The DAB-stained images were downsampled by a factor of 4 while preserving high resolution and then were processed using the thresholding module, adapted from Lim.^34^ Subsequently, an object classifier was employed to categorize cells equal to or above the adaptive threshold as positive and those below as negative, with classifier parameters described in Table 3. Once the classifier was applied, negative cells were displayed in blue, whereas positive cells appeared in red, and relevant data was stored for further postprocessing and visualization of the cell density maps.

**Table 3.** Cell classifier parameters.

Parameter	Value
Object classifier type	SimpleClassifier
Classification method	ClassifyByMeasurementFunction
Measurement used	DAB: Mean
Threshold	Adaptive
(Object) Filter	DETECTIONS_ALL

#### Saving additional raw data

Positive and negative cell density maps were generated using the classified nuclei with a search radius of 100 pixels (50 μm), which defines the surrounding area that contributes to the density value at each point. In addition, the annotation area in square millimeters and the total cell detections of each WSI were stored in a .txt file for further processing.

### Postprocessing in Python

In the Python script, data generated from QuPath was loaded and arranged to provide descriptive statistics and visualize results. Summary tables and box plots present findings by diagnosis, including positive and negative cell counts, total cell density, and Ki-67 LI. Additionally, the raw grayscale cell density maps generated by QuPath are loaded and processed by normalizing and visualizing them with a colormap, and the Ki-67 LI map is computed as the ratio of positive to total cells to improve interpretation.

### Validation

All slides were processed in a single round, and validation was performed through visual assessment and by performing a statistical comparison of the proposed tool to a benchmark research method.

### Visual assessment

The quality of tissue segmentation, cell segmentation and classification were visually assessed after the analysis by at least 1 nonpathologist, and; selected images with artifacts or doubtful tissue structure were revised by a pathologist. Based on the pathologist’s guidance, a total of 27 WSIs were excluded due to artifacts such as background staining, noise on the glass background, light exposure, out-of-focus images, and tissue containing a significant amount of bone fragments, affecting the stain vector estimation and leading to poor cell classification despite cells being well segmented.

### Comparative analysis

To evaluate the performance of the proposed tool, a comparative analysis was conducted with DeepLIIF,^23^ which was employed as a credible benchmark research method. In addition to the CBTN dataset, 2 open-access datasets were employed, comprising 30 neuroendocrine tumor tissue region images^35^ and 73 testicular seminoma tumor WSIs.^36^^,^^37^ The DeepLIIF framework was modified to enable batch processing of multiple WSIs for large-scale analysis across datasets. The analysis focused on statistically assessing the agreement between the 2 tools in terms of Ki-67 LI, positive and negative cell counts. Furthermore, images analyzed by the proposed tool and DeepLIIF were visually assessed.

### Statistical analysis

The nonparametric Kruskal-Wallis test was conducted to investigate whether a statistically significant correlation existed between the pediatric brain tumor types/families and Ki-67 LI, positive and negative cell density, and tumor descriptors. The significance level was set at a=0.05 and adjusted using the Bonferroni correction. The frameworks were compared using the intraclass correlation coefficient (ICC), Spearman’s correlation coefficient (*ρ*), both ranging between 0 and 1, and regression.

## RESULTS

**Figure 2.**
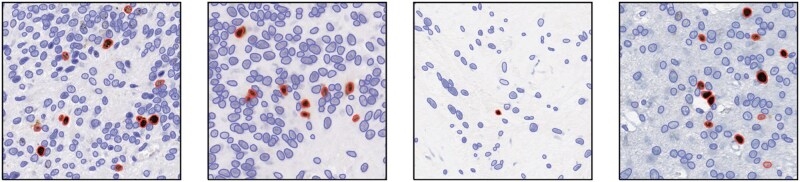
Zoomed-in representatives of Ki-67 WSIs. The cell segmentation and classification are performed by the Apache Groovy script. Ki-67 negative nuclei are stained and segmented in blue, and Ki-67-positive nuclei are stained brown and segmented with red. Abbreviations: Ki-67, Kiel 67; WSIs, whole slide images.

**Figure 3.**
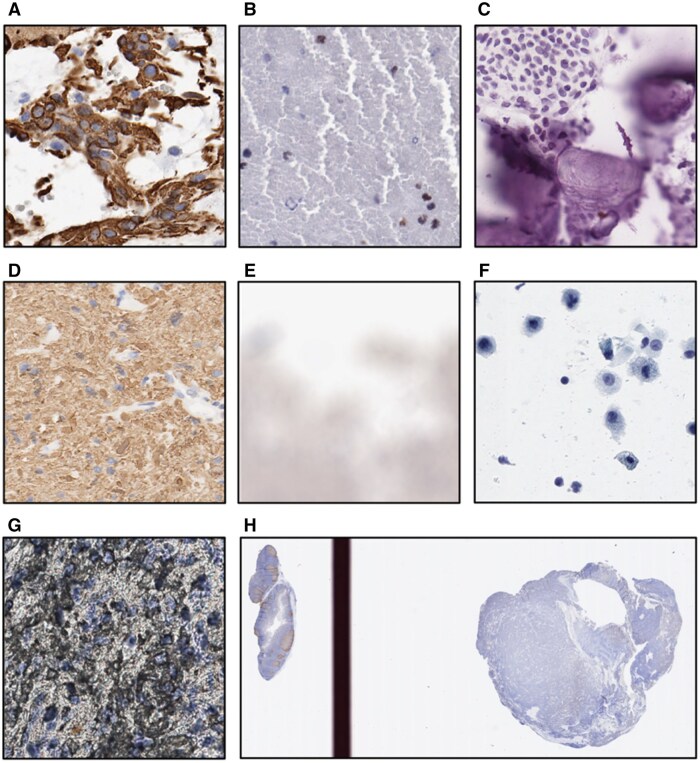
Example of cases in which cell classification was erroneous. (A) Stained cytoplasm and axons, (B) clumped erythrocytes, (C) bone fragments, (D) background staining, (E) out-of-focus WSI, (F) light exposure tissue, and (G) artifact during tissue preparation. (H) Example of a WSI with a control tissue sample on the left. Control tissue samples serve as a reference when tissue samples are examined. Abbreviation: WSI, whole slide image.

The results of the proposed tool’s Ki-67 LI, positive and negative cell counts on the CBTN dataset along with the validation are presented in the following sections. Examples of the cell segmentation and classification produced by the proposed tool are shown in Figure 2, and representative cases excluded due to artifacts along with a WSI with control tissue are depicted in Figure 3. Furthermore, artifacts, such as pen marks, did not interfere with tissue segmentation, and cell segmentation and classification.

### Ki-67 WSIs analysis

**Figure 4.**
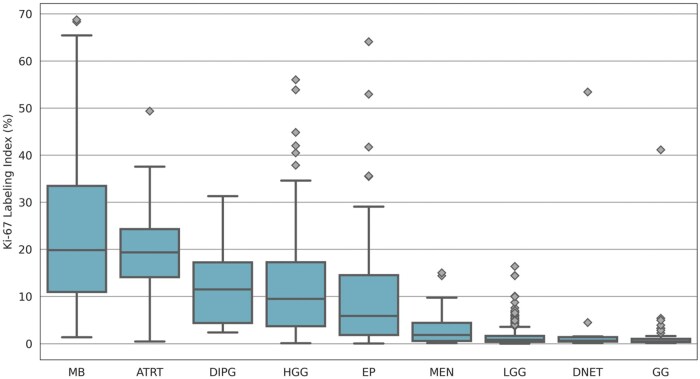
Box plot of Ki-67 LI across the tumor families/types at a WSI level. Abbreviations: Ki-67, Kiel 67; LI, labeling index; WSI, whole slide image.

In Figure 4, the box plot illustrates the distribution of Ki-67 LI. Table S1 provides the median, m±sd, maximum and minimum values across the tumor families/types. Medulloblastomas exhibited the highest median Ki-67 LI value (19.84), with a m±sd of 23.10±16.15 and a maximum of 68.75, ATRTs followed with a median of 19.36 and a m±sd of 20.48±11.2. DIPGs, HGGs, and ependymomas displayed Ki-67 LI values, with medians of 11.50, 9.50, and 5.88, respectively. Meningiomas had a lower Ki-67 LI, with a median of 1.84 and a m±sd of 3.37±3.92. The lowest Ki-67 LI values were observed in LGGs (median: 0.85), DNETs (median: 0.63), and gangliogliomas (median: 0.50). The statistical test suggested a significant correlation between Ki-67 LI and most tumor families/types, with DNET and meningioma not showing a statistically significant correlation.

**Figure 5.**
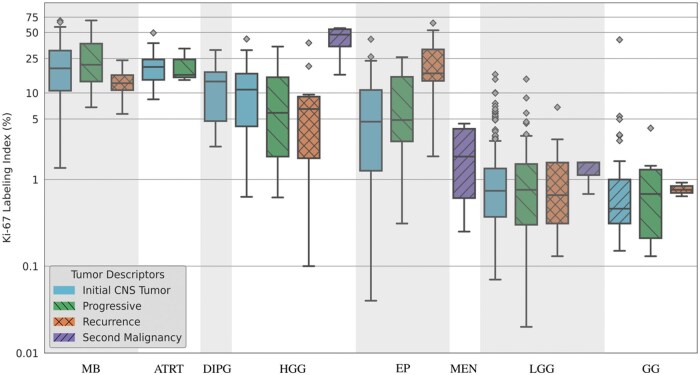
Logarithmic-scaled box plot of Ki-67 LI across the tumor families/types for each tumor descriptor (after the exclusions, initial CNS tumor: 400, progressive: 85, recurrence: 39, second malignancy: 12) at the WSI level. It should be noted that there is no one-to-one correspondence between subjects, diagnosis, and tumor descriptors. Abbreviations: Ki-67, Kiel 67; LI, labeling index; WSI, whole slide image.

Figure 5 describes a logarithmic-scaled box plot of Ki-67 LI across the tumor families/types for each tumor descriptor (after the exclusions, initial CNS tumor: 400, progressive: 85, recurrence: 39, second malignancy: 12). The distribution of Ki-67 LIs resembles a similar pattern observed in Figure 4 with no trend being visible within the tumor descriptors. Almost all combinations of tumor families/types and descriptors do not show a statistically significant association with Ki-67 LI, and the only notable significant correlations are observed for second malignancy HGGs and recurrent ependymomas, with the corresponding *P*-values shown in Table S2.

### Positive and negative cell densities

**Figure 6.**
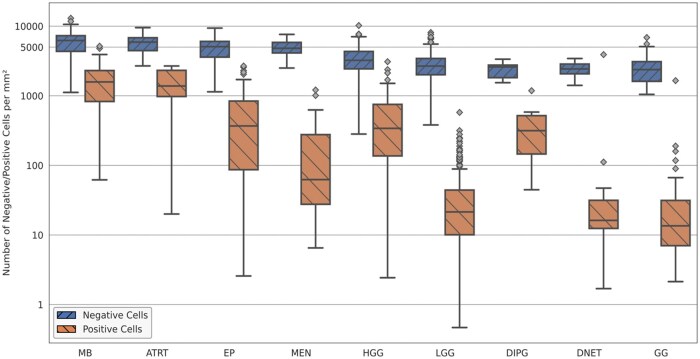
Logarithmic-scaled box plot of the number of the negative and positive cell density (number of negative/positive cells per mm^2^) across the tumor families/types at the WSI level. Abbreviation: WSI, whole slide image.

Figure 6 presents a logarithmic-scaled box plot illustrating the distribution of positive and negative cell densities (number of positive/negative cells per mm^2^) across the tumor families/types, with corresponding statistical values provided in Table S3. Among the tumors, medulloblastomas exhibited the highest positive and negative cell densities, with medians of 1582.44 cells per mm^2^ (m±sd: 1725.49±1098.42) and 6226.67 cells per mm^2^ (m±sd: 6148.9±2350.98), respectively. ATRT followed closely with a median of 1382.78 positive cells per mm^2^ (m±sd: 1522.89±807.12) and 5908.83 negative cells per mm^2^ (m±sd: 5907.57±1921.75). Ependymomas and HGGs demonstrated positive cell density medians of 368.35 and 340.63 cells per mm^2^, and negative cell density medians of 5080.68 and 3233.67 cells per mm^2^, respectively. DIPG showed slightly lower densities, with a median of 315.46 cells per mm^2^ for positive and 2598.08 cells per mm^2^ for negative cells. In contrast, meningiomas exhibited a relatively low positive cell density (median: 62.49 cells per mm^2^), but its negative cell density is comparatively higher (median: 4818.41 cells per mm^2^), exceeding those of HGGs and DIPGs. The lowest positive and negative cell densities were noticed in LGGs, DNETs, and gangliogliomas. The statistical analysis demonstrated a significant correlation between positive/negative cell density and most of the tumor families/types, except for DNETs and DIPGs, while no significant correlation was noted between positive cell density and meningiomas, and between negative cell density and HGGs.

### Density maps

**Figure 7.**
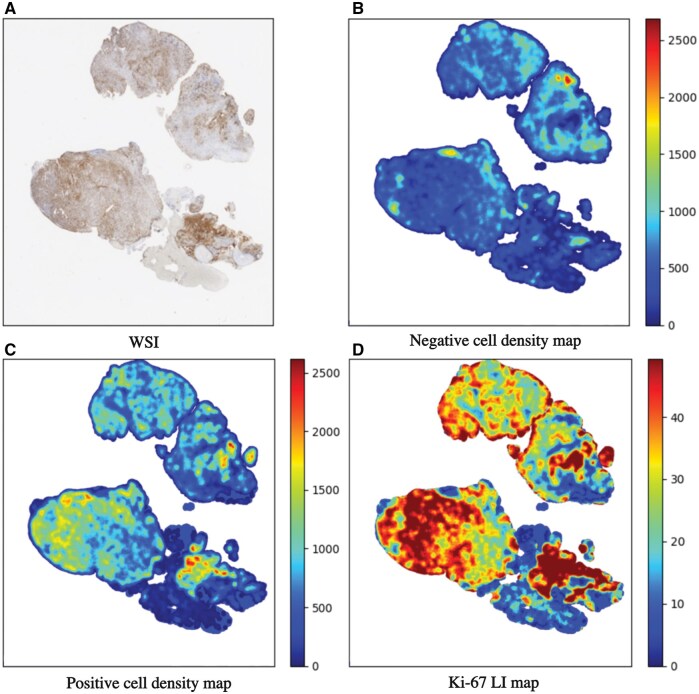
Examples of (A) WSI for an ATRT tumor with overall Ki-67 LI=49.35 along with the corresponding (B) negative and (C) positive cell density maps, and (D) the Ki-67 LI map. Abbreviations: ATRT, atypical teratoid rhabdoid tumor; Ki-67, Kiel 67; LI, labeling index; WSI, whole slide image.

The negative and positive cell density maps for each slide were generated by QuPath, which were processed and visualized by the Python script, with the Ki-67 LI map also computed. Figure 7 provides an example of cell density and Ki-67 LI maps of a subject diagnosed with ATRT with a Ki-67 LI of 49.35. The negative cell density map visualizes the spatial distribution of negatively stained cells in the tissue, with cooler colors (eg, blue) representing areas with lower negative cell density and warmer colors (eg, red) indicating regions with higher negative cell density. The color bar associated with this map provides a scale of the density values, helping to interpret the intensity of negative cell density across the tissue. The positive cell density map similarly shows the distribution of positively stained cells, with warmer colors representing higher densities of positive cells. The Ki-67 LI map represents the ratio of positively stained cells to the total number of cells, with warmer colors demonstrating a higher ratio of positive cells and cooler colors showing a lower ratio of positive cells. The color bar provides a scale for the LI, allowing for a clear interpretation of the proportion of positive cells in different tissue regions.

### Validation

**Figure 8.**
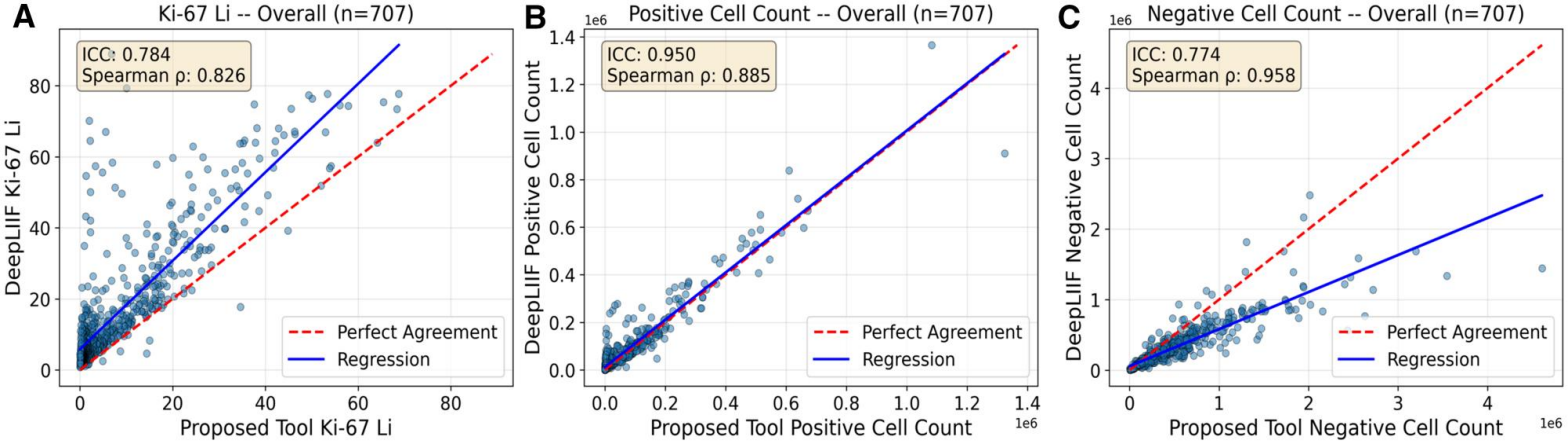
Scatterplots comparing (A) Ki-67 LI, (B) positive , and (C) negative cell counts between the proposed tool and DeepLIIF for the CBTN dataset. The corresponding ICC and Spearman’s *ρ* values are reported, with regression lines and lines of perfect agreement illustrated. Abbreviations: CBTN, Children’s Brain Tumor Network; ICC, intraclass correlation coefficient; Ki-67, Kiel 67; LI, labeling index.

The proposed tool and DeepLIIF demonstrated comparable performance using the CBTN dataset as shown in Figure 8. More specifically, the Ki-67 LI exhibited moderate to high agreement (Spearman’s *ρ*=0.826, ICC=0.784), with DeepLIIF tending to overestimate the LI values. Positive cell counts demonstrated very strong agreement (Spearman’s *ρ*=0.885, ICC=0.950), a pattern being consistent across most tumor types and families in the CBTN dataset as depicted in Figure S2, while the agreement for negative cell was moderate to high (Spearman’s *ρ*=0.958, ICC=0.774). Systematic differences were observed between the 2 methods, with DeepLIIF’s tendency to count fewer negative cells, as closely clustered cells are often segmented as a single cell. In contrast, the proposed method provided more accurate segmentation of round and elongated cells, leveraging StarDist’s architecture, which is designed to resolve overlapping nuclei. Additionally, DeepLIIF was more sensitive in detecting and segmenting nontumor elements, such as erythrocytes and tissue background, often falsely labeling them as positive or negative cells. Therefore, the combination of undersegmentation of clustered negative nuclei and increased sensitivity to nontumor elements contributed to the inflated LI values provided by DeepLIIF.

Similar trends were observed in the 2 additional datasets. In neuroendocrine tumor images, positive cell counts showed moderate agreement (Spearman’s *ρ*=0.708, ICC=0.710), while negative cell counts demonstrated low agreement (Spearman’s *ρ*=0.329, ICC=0.078), resulting in moderate agreement for Ki-67 LI (Spearman’s *ρ* =0.377, ICC=0.648). In testicular seminomas, agreement across all metrics was consistently moderate, with positive cell counts (Spearman’s *ρ*=0.650, ICC=0.530), negative cell counts (Spearman’s *ρ*=0.840, ICC=0.638), and Ki-67 LI (Spearman’s *ρ*=0.644, ICC=0.421).

Across all datasets, the results indicate that the proposed tool achieves comparable performance to DeepLIIF, although certain limitations were observed in each (Table S4, Figures 8 and S1-S6). Representative examples illustrating the disparities between the 2 methods are provided in Figure S6.

## DISCUSSION

In this study, an Apache Groovy code script for QuPath was developed to automatically calculate the Ki-67 LI in pediatric brain tumors for research purposes. Additionally, Python-based algorithms were added to generate summary tables and graphs of the Ki-67 scores and visualize the density maps. The proposed tool offers several benefits over existing approaches, including integration with QuPath, a widely used digital pathology platform that provides a versatile set of tools, which could be used after the automated analysis to perform more detailed examination and annotation of the tissue. For example, after the tissue images are processed using the proposed tool, users can interact with the WSI by selecting regions of interest to extract localized LIs, ensuring that measurements correspond only to tumor regions. Slide-level analysis is supported along with batch processing of large datasets, and adaptable thresholds are implemented for tissue segmentation and cell classification to reflect stain variability and other image-specific features. Additionally, the StarDist framework was employed reliably outlining object boundaries and providing more accurate segmentation of round and elongated shaped cells. This method provides a higher generalizability over the manual threshold setting in QuPath.^38^ The Apache Groovy script in QuPath has the potential to be further adapted for automated scoring of other stains and cancer types but the process should be evaluated.

Postanalysis is supported by density maps, allowing pathologists to simultaneously annotate and examine the Ki-67 LI levels in specific regions of interest. The generated Ki-67 LI maps are practical for highlighting regions with increased positive cell density, simplifying the analysis compared to using only the negative and positive cell density maps. These maps could be used to identify Ki-67 hot spots in WSIs or be compared with explanatory maps (or heatmaps) generated by deep learning models to understand how the model’s attention is localized within tissue regions,^39^ as demonstrated in our prior study.^40^

The Ki-67 LI values derived from the analysis were aligned with the established oncological consensus and the statistical analysis indicated that the Ki-67 LI is a significant marker for distinguishing between some of tumor families/types. Specifically, medulloblastomas and ATRTs exhibited considerably higher Ki-67 LI values compared to other tumor families/types, reflecting their higher malignancy. A broad range of Ki-67 LI values is observed in medulloblastomas, possibly reflecting the presence of multiple molecular subgroups. Similarly, the ependymoma cases are related to high Ki-67 LI, however grade 2 and grade 3 tumors might not be differentiated in the CBTN dataset, contributing to the variability in the Ki-67 LI. DIPGs are typically associated with high Ki-67 LI values, with the definitive diagnosis often requiring molecular testing, as morphological features alone may be insufficient to confirm malignancy. HGGs are generally associated with Ki-67 LI values above 10, with some borderline cases being challenging to classify as low Ki-67 LI values observed in HGGs could represent a mixture of low- and high-grade areas. The meningiomas, LGGs, DNETs, and gangliogliomas are typically considered low-malignancy tumors, consistent with low Ki-67 LI values in this study. Regarding the relationship between the Ki-67 LI of the tumor families/types and the descriptors, no meaningful insights could be extracted. The absence of a one-to-one correspondence between subjects, tumor family/type, and descriptors limited the feasibility and interpretability of such analysis.

The proposed tool and DeepLIIF demonstrated comparable performance, although some differences were observed. Specifically, DeepLIIF counted fewer negative cells, as closely clustered nuclei were often segmented as a single cell, whereas the proposed tool benefits from the ability of StarDist to resolve overlapping or clustered cells. In addition, DeepLIIF had a higher tendency in falsely segmented nontumor cells compared with the proposed tool. Consequently, in some cases these factors led to disparities in the Ki-67 LI between the two tools.

Regarding the limitations of the study, the experiments were primarily limited by the lack of ground truth, that is, confirmed Ki-67 LI values and positive and negative cell counts, which were not provided with the CBTN dataset, and this restricted quantitative validation of the results. The most accurate ground truth is based on the manual cell annotation by an expert pathologist. However, it is reported that Ki-67 LI assessment shows a considerable interobserver variability, scorer training and calibration is recommended to standardize the process.^17^ Moreover, this process should be performed on large areas of the slide, which makes it impractical considering the scale and variability of the data included. Another limitation is that the excluded images consisted of cases with severe tissue preparation artifacts, which hindered the accurate visual assessment by a pathologist, as well as cases with visible artifacts, such as bone fragments, which did not significantly impact tissue and cell segmentation but disrupted the stain vector estimation, leading to misleading cell classification. While these cases were relatively few, they still limited the full potential of an automated analysis. Additionally, in rare cases, in which nontumor cells, such as erythrocytes, were mistakenly segmented and classified as negative cells, their impact on the Ki-67 LI was minimal, as independent assessments showed that Ki-67 LI in the manually selected regions closely matched the values generated by the algorithm. Accurately distinguishing tumor from non-tumor cells remains a challenge that is not fully addressed by the current study. In some cases, lightly stained nuclei were not segmented, although manual review showed that minor undetected nuclei had minimal impact on the Ki-67 LI. Per-tile normalization and a detection threshold of 0.25 were applied across the CBTN dataset to accommodate local variations in staining intensity, improving the cell detection across lightly and heavily stained regions. As described in the “Cell Segmentation and Classification” section, adjusting the threshold involved a tradeoff, with lower values increasing sensitivity but potentially introducing false positives and higher computational load, while higher values risked missing lightly stained or overlapping nuclei. Users could adjust the detection threshold if needed to improve the segmentation of lightly or heavily stained cells, as well as in areas with closely spaced nuclei, even though StarDist is designed to resolve overlapping nuclei by default. Therefore, future development should focus on refining tumor cell counting accuracy and improving the exclusion of nontumor cells. This would best be possible by using double staining or cytology classification algorithms.^5^ Finally, access to the tumor grades of meningioma and ependymoma, and the molecular subgroups of medulloblastoma, as well as slide-level diagnosis, would have allowed for a more precise analysis.

## CONCLUSION

We report the successful development and evaluation of an automated pipeline for quantifying the Ki-67 LI across various pediatric brain tumor families/types, utilizing an Apache Groovy script in QuPath and a Python script for postprocessing. The obtained pediatric brain tumor Ki-67 LI values were in accordance with the pathology and oncology consensus, though a few outliers were observed. The results obtained with the proposed method were comparable to those of DeepLIIF, which showed a tendency to inflate Ki-67 LI values. Additionally, the cell density and Ki-67 LI maps provide a spatial analysis of cell proliferation in pediatric brain tumors, facilitating the identification of areas with high Ki-67 expression or providing automatic annotation for analyzing the explanatory maps of the deep learning models. Overall, these scripts provide an accessible tool for automatically analyzing large-scale Ki-67 WSI data in research settings, with the potential for adaptation to other cancer types and stains.

## Supplementary Material

nlaf163_Supplementary_Data

## Data Availability

The open-access dataset used in this study was obtained from the Children’s Brain Tumor Network (https://cbtn.org). The dataset for neuroendocrine and testicular seminoma are open-access and are available at https://github.com/cialab/neuroendocrine_ and https://zenodo.org/records/11218961, respectively. The code and detailed instructions for execution linked to this study are available in the GitHub repository: https://github.com/Christoforos-Spyretos/QuPath-Automatic-Cell-Detection-for-Ki-67-WSIs
