# Supplementary material for: Quantification of Ki-67 labeling index in pediatric brain tumor immunohistochemistry images
Source: J Neuropathol Exp Neurol. 2026 Mar 10;85(5):475–86. doi: 10.1093/jnen/nlaf163 (PMC13127889; doi:10.1093/jnen/nlaf163)
Supplement: nlaf163_Supplementary_Data [file nlaf163_supplementary_data.docx]

# Supplementary Material

**Table S1:** Summary of the number of the subjects and WSIs included in the results, median (range), mean ± standard deviation of the Ki-67 LI for each tumor family/type. P-values shown in bold indicate statistically significant differences in Ki-67 LI between tumor families/types, using Kruskal-Wallis test with significance level adjusted using Bonferroni correction $a=0.05 / 9=0.00556$.

| **Tumor Family/type** | **No. of Subjects**  **(Male/Female)** | **No. of WSI** | **Median**  **[Range]** | **Mean ± Std** | **p-value** |
| --- | --- | --- | --- | --- | --- |
| MB | 80  (45/35) | 85 | 19.84  [1.36, 68.75] | 23.10 ± 16.15 | **<0.001** |
| ATRT | 20  (13/7) | 21 | 19.36  [0.46, 49.35] | 20.48 ± 11.2 | **<0.001** |
| DIPG | 10  (7/3) | 11 | 11.50  [2.40, 31.30] | 12.69 ± 9.19 | **0.005** |
| HGG | 88  (48/40) | 102 | 9.50  [0.10, 56.02] | 12.78 ± 11.93 | **<0.001** |
| EP | 70  (44/26) | 88 | 5.88  [0.04, 64.09] | 10.24 ± 11.79 | **<0.001** |
| MEN | 23  (13/10) | 33 | 1.84  [0.12, 15] | 3.37 ± 3.92 | 0.267 |
| LGG | 257  (140/117) | 291 | 0.85  [0.02, 16.37] | 1.56 ± 2.18 | **<0.001** |
| DNET | 13  (10/3) | 14 | 0.63  [0.12, 53.4] | 4.77 ± 14.04 | 0.038 |
| GG | 50  (28/22) | 63 | 0.50  [0.13, 41.12] | 1.59 ± 5.18 | **0.005** |
| **Total** | **611**  **(327/284)** | **708** | **-** | **-** | **-** |

**Table S2:** Results of the Kruskal-Wallis test assessing the statistical correlation between Ki-67 LI and tumor descriptors (initial CNS tumor, progressive, recurrence, second malignancy) within each tumor family/type at a significance level a=0.05 . P-values shown in bold indicate statistically significant correlation between the Ki-67 LI and tumor families/types, using Kruskal-Wallis test with the significance level was adjusted using a Bonferroni correction based on the number of comparisons within each tumor family/type.

| **Tumor Family/type** | **Tumor Descriptor** | **No. of Subjects**  **(Male/Female)** | **No. of WSI** | **p-value** |
| --- | --- | --- | --- | --- |
| MB | Initial CNS Tumor | 70  (41/29) | 70 | 0.927 |
|  | Progressive | 2  (1/1) | 4 | 0.667 |
|  | Recurrence | 4  (1/3) | 4 | 0.344 |
| ATRT | Initial CNS Tumor | 16  (6/10) | 16 | 0.947 |
|  | Progressive | 3  (-/3) | 3 | 0.848 |
| HGG | Initial CNS Tumor | 52  (24/28) | 54 | 0.680 |
|  | Progressive | 17  (8/9) | 21 | 0.232 |
|  | Recurrence | 8  (5/3) | 10 | 0.291 |
|  | Second Malignancy | 4  (-/4) | 4 | **0.0048** |
| EP | Initial CNS Tumor | 47  (30/17) | 54 | 0.346 |
|  | Progressive | 9  (5/4) | 10 | 0.989 |
|  | Recurrence | 8  (5/3) | 8 | **0.0072** |
| LGG | Initial CNS Tumor | 175  (91/84) | 185 | 0.971 |
|  | Progressive | 47  (26/21) | 53 | 0.973 |
|  | Recurrence | 16  (9/7) | 17 | 0.842 |
|  | Second Malignancy | 3  (2/1) | 3 | 0.310 |
| GG | Initial CNS Tumor | 31  (16/15) | 41 | 0.919 |
|  | Progressive | 7  (5/2) | 7 | 0.877 |
|  | Recurrence | 3  (2/1) | 3 | 0.461 |
| MEN | Second Malignancy | 5  (1/4) | 5 | 1 |
| DIPG | Initial CNS Tumor | 9  (2/7) | 10 | 1 |
| **Total** | **-** | **536**  **(280/256)** | **582** | **-** |

**Table S3:** Summary of the median (range), mean ± standard deviation of the positive and negative cell density (cells per mm2) for each tumor family/type. P-values shown in bold indicate statistically significant correlation between negative/positive cell density (number of negative/positive cells per mm^2^) and tumor families/types, using Kruskal-Wallis test with significance level adjusted using Bonferroni correction α = 0.05/9 = 0.00556.

| **Tumor Family/type** | **Negative Cell Density** | | | **Positive Cell Density** | | |
| --- | --- | --- | --- | --- | --- | --- |
|  | **Median**  **[Range]** | **Mean ± Std** | **p-value** | **Median**  **[Range]** | **Mean ± Std** | **p-value** |
| MB | 6226.67  [12995.28, 1119.45] | 6148.9 ± 2350.98 | **<0.001** | 1582.44  [61.97, 5159.88] | 1725.49 ± 1098.42 | **<0.001** |
| ATRT | 5908.83  [9526.94, 2688.03] | 5907.57 ± 1921.75 | **<0.001** | 1382.78  [20.02, 2682.21] | 1522.89 ± 807.12 | **<0.001** |
| EP | 5080.68  [9381.84, 1141.03] | 4995.47 ± 1756.61 | **<0.001** | 368.35  [2.58, 2678.02] | 582.50 ± 649.80 | **<0.001** |
| HGG | 3233.67  [10223.09, 281.82] | 3542.78 ± 1701.45 | 0.705 | 340.63  [2.43-3088.62] | 515.39 ± 540.29 | **<0.001** |
| DIPG | 2598.08  [3357.26, 1543.41] | 2423.4 ± 596.65 | 0.017 | 315.46  [44.68, 1183.52] | 373.28 ± 328.61 | 0.051 |
| MEN | 4818.41  [7622.89, 2503.9] | 5044.7 ± 1285.82 | **<0.001** | 62.49  [6.52, 1212.32] | 196.53 ± 280.65 | 0.695 |
| LGG | 2674.58  [8107.55, 381.12] | 2828.13 ± 1225.46 | **<0.001** | 21.45  [0.47, 577.11] | 41.20 ± 59.05 | **<0.001** |
| DNET | 2431.76  [3357.26, 1411.35] | 2453.78 ± 638.27 | 0.009 | 16.21  [1.7, 3907.71] | 302.57 ± 1037.99 | 0.018 |
| GG | 2376.8  [6868.12, 1048.48] | 2540.76 ± 1205.08 | **<0.001** | 13.54  [2.14, 1652.21] | 50.93 ± 207.85 | **<0.001** |

**Table S4:** Spearman’s correlation coefficients (ρ) comparing Ki-67 LI, positive and negative cells between the proposed tool and DeepLIIF across three datasets.

| **Dataset** | **Number of Samples** | **Spearman’s ρ Ki-67 LI** | **Spearman’s ρ Positive Cells** | **Spearman’s ρ Negative Cells** |
| --- | --- | --- | --- | --- |
| Pediatric brain tumor (CBTN) | 708 WSIs | 0.826 | 0.885 | 0.958 |
| Neuroendocrine | 30 tissue regions | 0.688 | 0.708 | 0.329 |
| Testicular Seminoma | 73 WSIs | 0.644 | 0.650 | 0.840 |

**
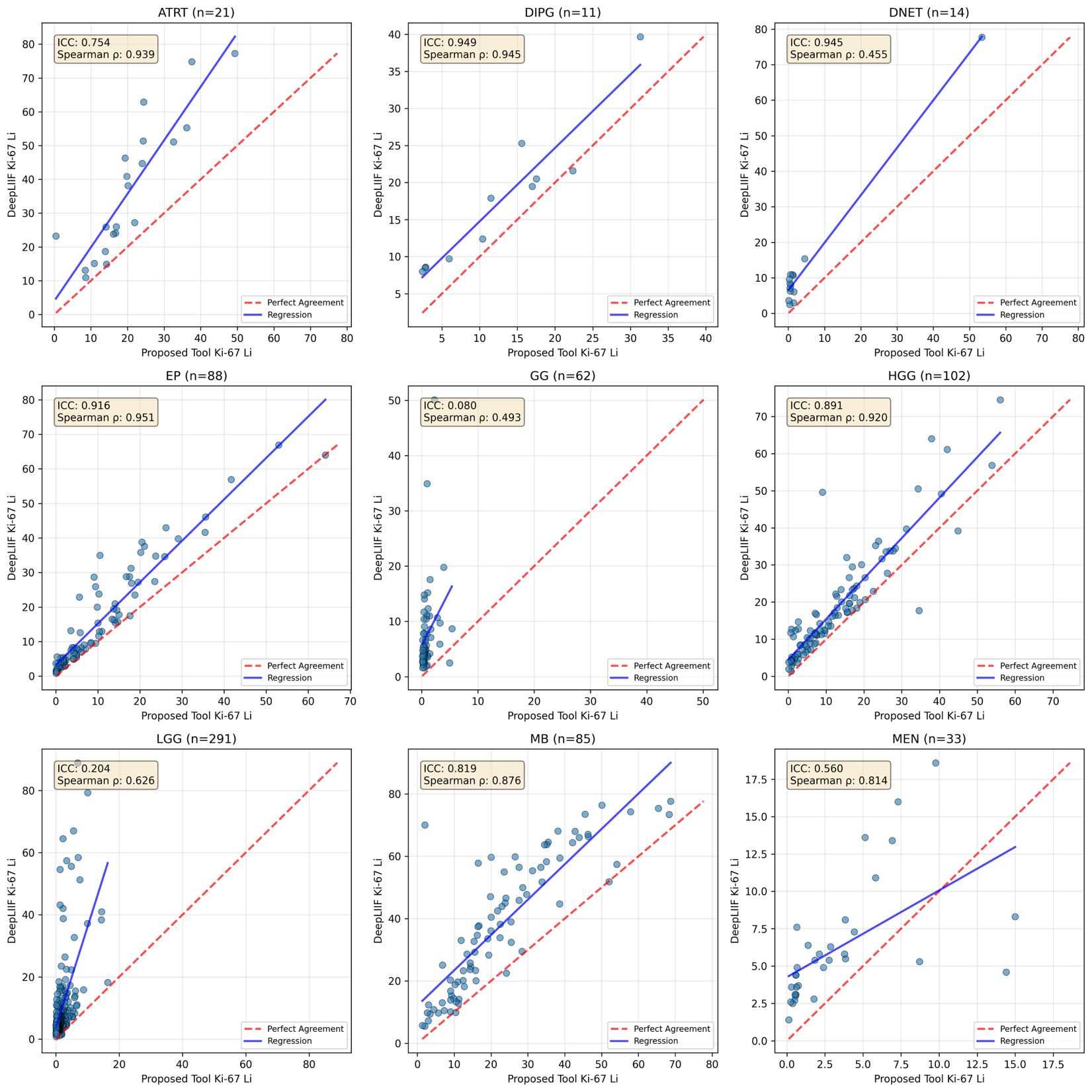
**

**Figure S1:** Scatterplots comparing label-wise Ki-67 LI between the proposed tool and DeepLIIF for the CBTN dataset. The corresponding ICC and Spearman’s ρ values are reported, with the regression line and the line of perfect agreement (dashed line) illustrated.


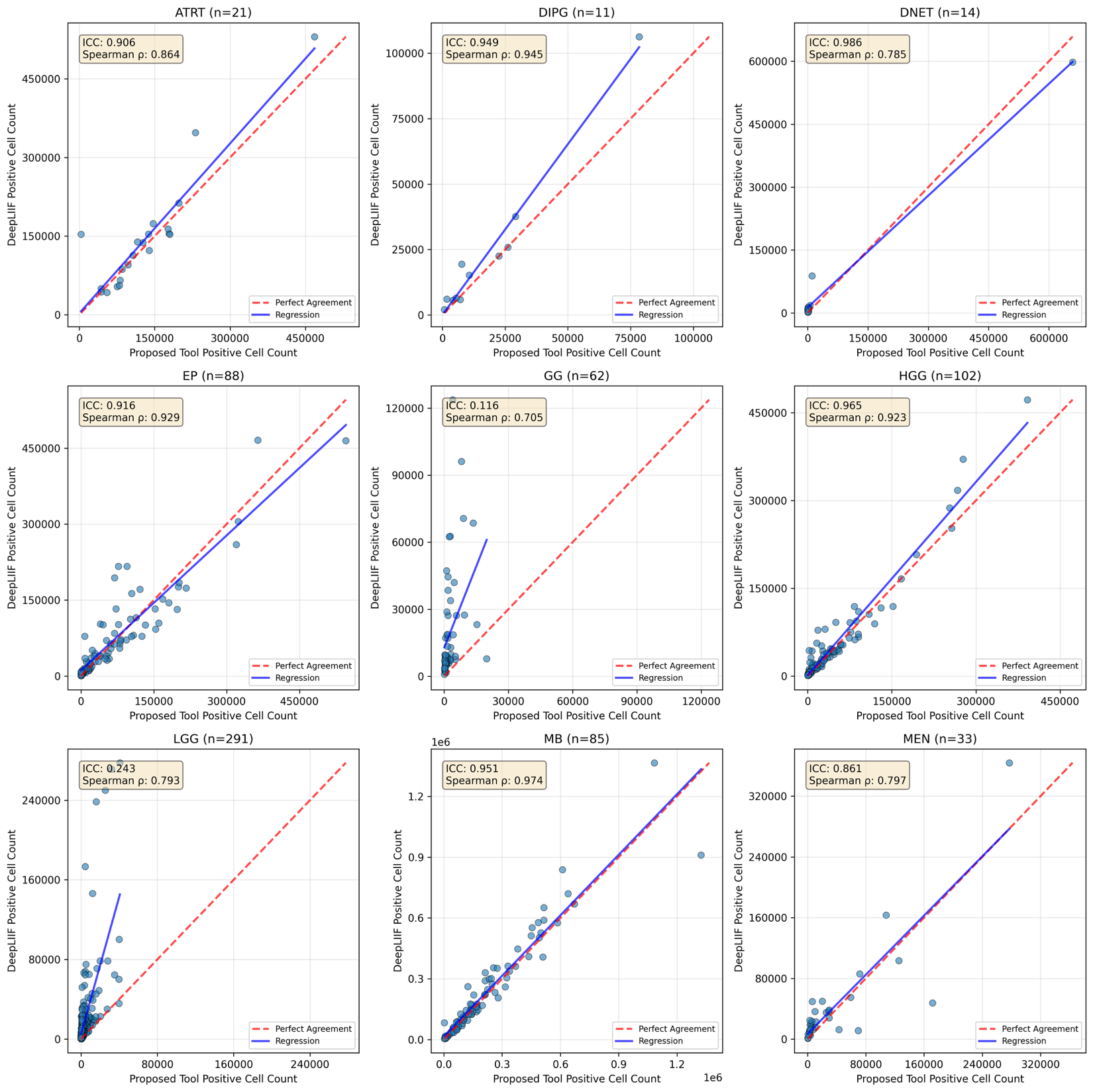


**Figure S2:** Scatterplots comparing label-wise positive cell counts between the proposed tool and DeepLIIF for the CBTN dataset. The corresponding ICC and Spearman’s ρ values are reported, with the regression line and the line of perfect agreement (dashed line) illustrated.


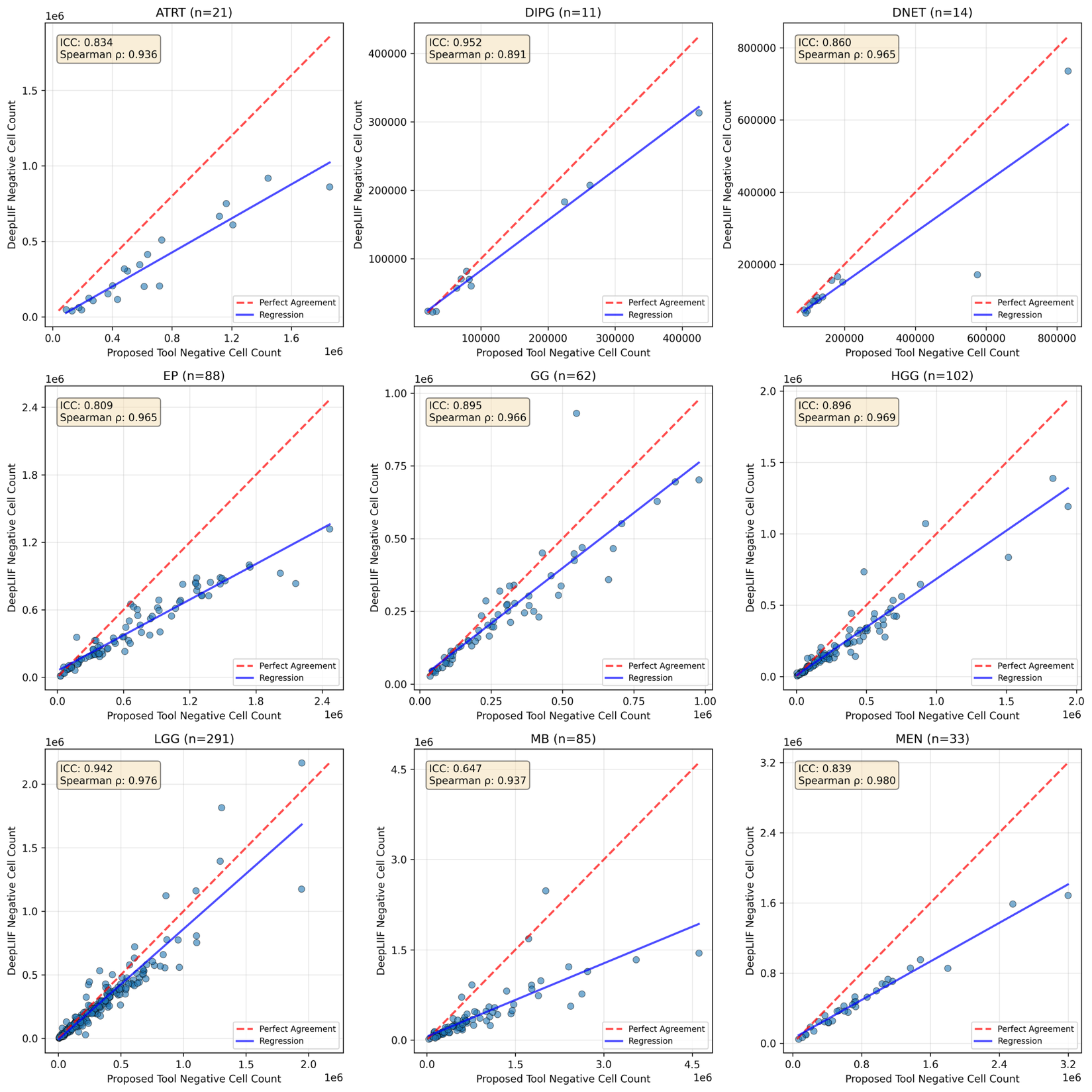


**Figure S3:** Scatterplots comparing label-wise negative cell counts between the proposed tool and DeepLIIF for the CBTN dataset. The corresponding ICC and Spearman’s ρ values are reported, with the regression line and the line of perfect agreement (dashed line) illustrated.


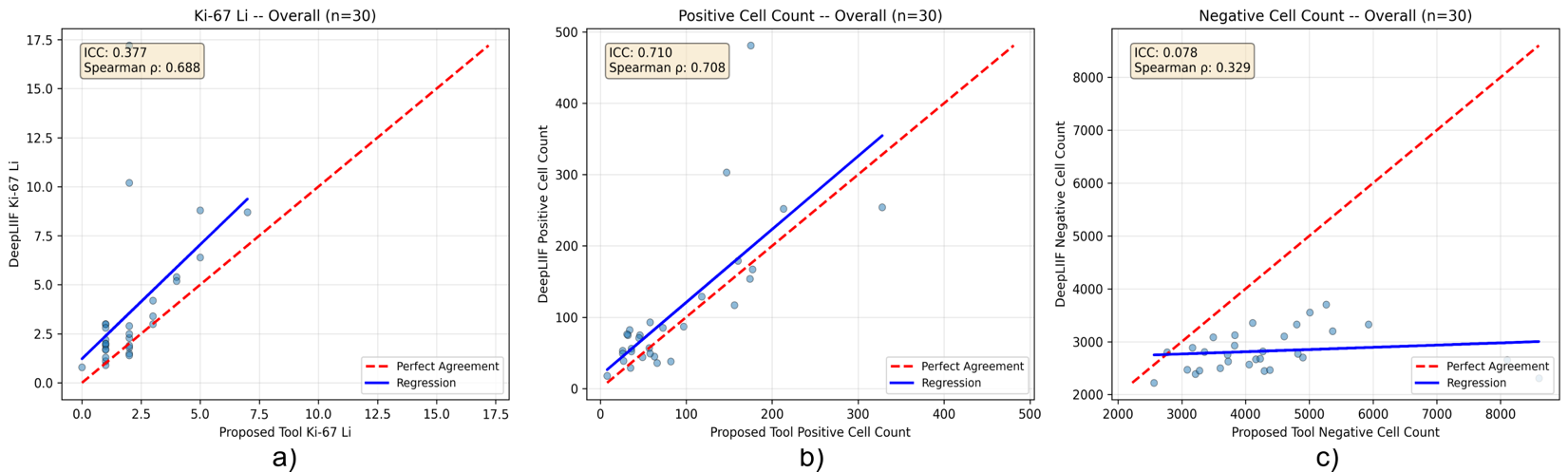


**Figure S4:** Scatterplots comparing a) Ki-67 LI, b) positive, and c) negative cell counts between the proposed tool and DeepLIIF for the neuroendocrine dataset. The corresponding ICC and Spearman’s ρ values are reported, with the regression line and the line of perfect agreement (dashed line) illustrated.


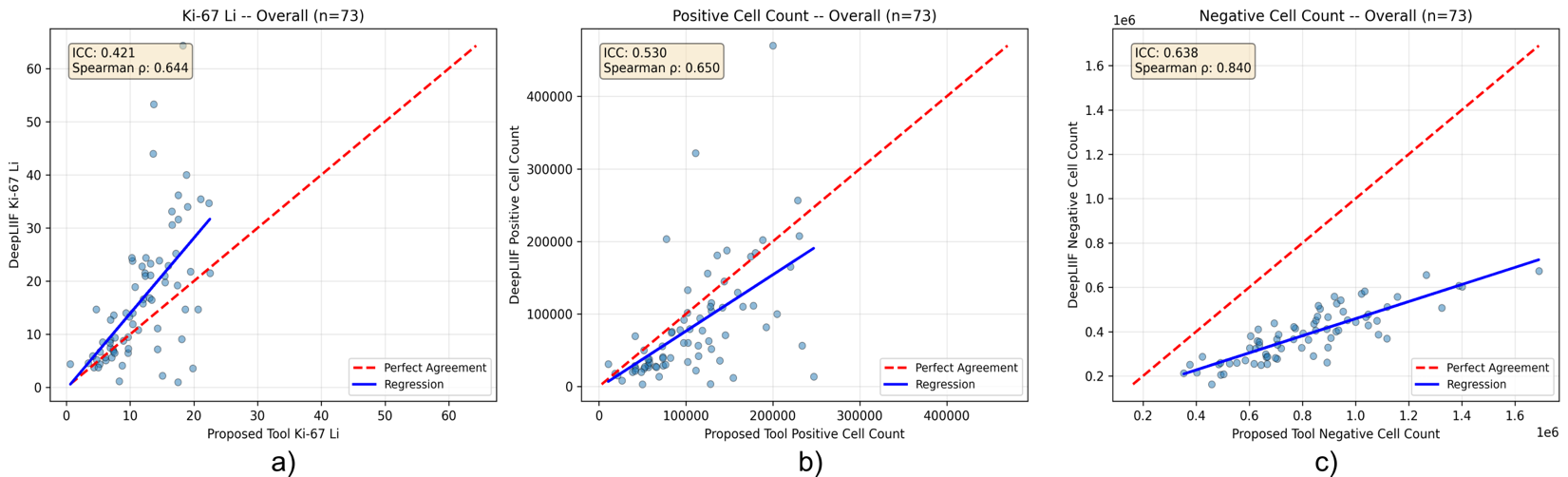


**Figure S5:** Scatterplots comparing a) Ki-67 LI, b) positive, and c) negative cell counts between the proposed tool and DeepLIIF for the testicular seminoma dataset. The corresponding ICC and Spearman’s ρ values are reported, with the regression line and the line of perfect agreement (dashed line) illustrated.


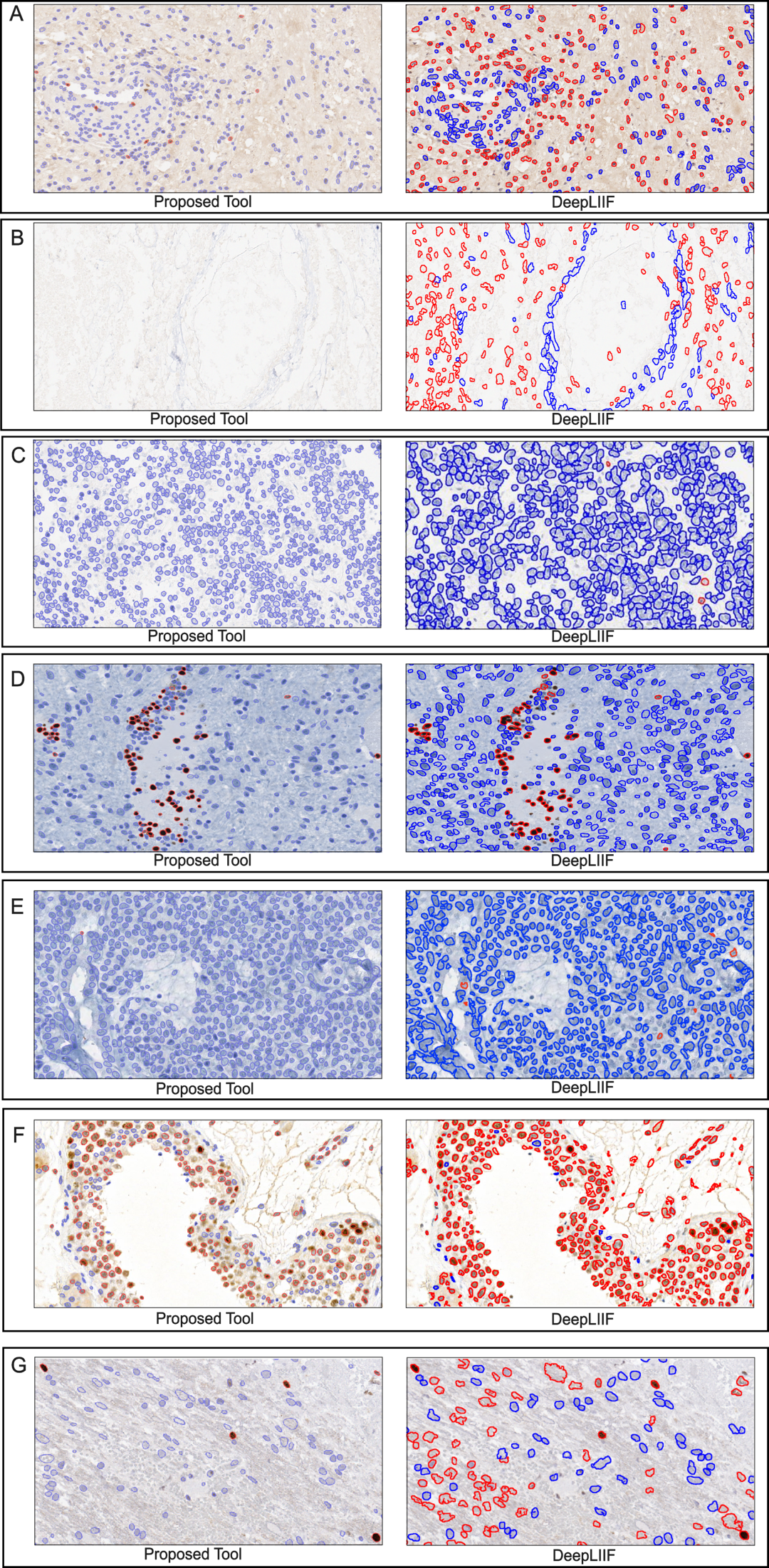


**Figure S6:** Examples of cell segmentation and classification results obtained using the proposed tool (left column) and DeepLIIF (right column) across different datasets. Panels A–D and G correspond to the CBTN, panel E to the neuroendocrine and panel F to testicular seminoma dataset. In panels A, B, and F, non-tumor elements such as erythrocytes and brown tissue background were more frequently detected and labeled as positive or negative cells by DeepLIIF, resulting in inflated Ki-67 LI values. In panels C, D, and E, closely clustered negative cells were often segmented as single large cells by DeepLIIF, leading to reduced Ki-67 LI values. In panel G, both tools falsely segmented background tissue as cells.
